# Red Blood Cell Depletion in Patients With Hyperferritinemia: Comparison of Two Apheresis Systems

**DOI:** 10.1002/jca.70097

**Published:** 2026-02-22

**Authors:** Heidrun Neureiter, Nadja Schroeder, Orkan Kartal, Sandra Laner‐Plamberger, Wanda Lauth, Eva Rohde, Christoph Grabmer

**Affiliations:** ^1^ Department for Transfusion Medicine University Hospital of Salzburg (SALK), Paracelsus Medical University (PMU) Salzburg Salzburg Austria; ^2^ Team Biostatistics and Big Medical Data, IDA Lab Salzburg, PMU Salzburg Salzburg Austria; ^3^ Research Programme Biomedical Data Science, PMU Salzburg Salzburg Austria

**Keywords:** Alyx, dysmetabolic iron overload syndrome, erythrocyte apheresis, extracorporeal volume, hereditary hemochromatosis, iron overload, spectra Optia, Spectra Optia

## Abstract

Red blood cell (RBC) apheresis is, alongside phlebotomy, a standard treatment for iron overload in hereditary hemochromatosis (HH). We compared the serum ferritin (SF) reduction, process parameters, duration, and side effects of two apheresis systems: Spectra Optia apheresis system (Optia) and Alyx apheresis collection system (Alyx). Forty‐three patients were RBC depleted with one of the two separators, Optia or Alyx. In total, 186 procedures were performed. The main diagnoses were HH (*n* = 20) and dysmetabolic iron overload syndrome (DIOS) (*n* = 21). Around two thirds of the procedures were done with Optia (*n* = 143) and one third with Alyx (*n* = 43). A mean volume of 405 and 442 mL of RBCs was withdrawn per single treatment with the Optia and Alyx, respectively. The procedure took 12 min (Optia) versus 26 min (Alyx) with a hematocrit (Hct) reduction of 5% versus 7.5% (*p* < 0.001). The SF reduction 3 weeks after RBC depletion was not significantly different between the two systems. The amount of anticoagulant used with the Optia was almost half of what was used with the Alyx (63 mL compared to 123 mL). There were no significant adverse events. The advantages of the Alyx include the lower cost and the easy portability. The Optia, on the other hand, has a shorter procedure time, a smaller extracorporeal volume, a lower anticoagulant consumption, a lower rate of complications, and allows a precise Hct adjustment.

## 1 Introduction

Hyperferritinemia, a condition of elevated ferritin levels in the blood, can be caused by various factors. One of the most common conditions is hereditary hemochromatosis (HH). This genetic disorder is characterized by an excess of body iron due to increased absorption of iron in the duodenum, usually caused by a Cys282Tyr mutation in the HFE gene on chromosome 6. This results in a progressive accumulation of iron in various organs, which can lead to significant health problems [1].

Another common cause of elevated ferritin is the dysmetabolic iron overload syndrome (DIOS) [2]. This is characterized by a mild increase in both liver and body iron stores and is associated with various components of metabolic syndrome [3].

The clinical objective in the management of patients with hyperferritinemia is to reduce ferritin levels in order to prevent the complications associated with iron overload. There are several therapeutic approaches, including phlebotomy and RBC apheresis. Both methods remove excess iron from the body by reducing RBCs and thus hemoglobin (Hb), where most of the body's iron is stored. Phlebotomy removes about 450 mL of whole blood, whereas RBC apheresis only removes the RBCs from the body by centrifugation, returning plasma, platelets, and white blood cells (WBCs). RBC depletion allows more RBCs, and therefore iron, to be removed from the body in a shorter time.

RBC apheresis is now a standard treatment for HH, next to phlebotomy. RBC apheresis is more efficient and halves the number of treatment procedures for all patients [1]. The guidelines of the American Society for Apheresis (ASFA) recommend the use of RBC apheresis as a first‐line therapy for HH [4].

In contrast to HH, the treatment recommendations for DIOS have changed over the years and currently phlebotomy or RBC apheresis cannot be considered a valuable option in DIOS patients [3, 5].

In 2015, we performed RBC apheresis for the first time with the Spectra Optia Apheresis System (Terumo BCT, Lakewood, USA) [6]. For cost reasons, we introduced in addition to the Spectra Optia System (Optia) the Alyx apheresis System (Fresenius Kabi, Bad Homburg, Germany), which is usually used for blood donation. This study compares the two RBC depletion systems in terms of the reduction of SF, their process parameters and duration, and the side effects experienced during and after treatment.

## 2 Materials and Methods

### 2.1 Patients

Between December 2013 and January 2022, a total of 43 patients (42 males and one female) with hyperferritinemia were iron‐depleted at least once by RBC apheresis. The main diagnoses were HH (*n* = 20) and DIOS (*n* = 21). Other diagnoses were secondary polyglobulia (*n* = 1) and iron overload due to excessive iron substitution in Crohn's disease (*n* = 1) as shown in Table 1.

**TABLE 1:** Patient characteristics.

	Optia	Alyx
Patients	36	20
Number of procedures	143	43
Age in years (mean ± SEM)	50.61 ± 2.18	54.8 ± 2.7
Sex M/F (*n*)	35/1	20/0
Patients diseases (*n*)		
HH	17	13
DIOS	13	6
Hyperferritinemia	4	1
Secondary polyglobulia	1	0
Iatrogenic iron overload	1	0
Weight in kg (mean ± SEM)	91.89 ± 2.42	93.8 ± 3.74
Height in cm (mean ± SEM)	180.61 ± 1.15	181.25 ± 1.74
Total blood volume in mL (mean ± SEM)	5729.64 ± 102.88	5838.95 ± 155.88

Abbreviations: DIOS, dysmetabolic iron overload syndrome; HH, hereditary hemochromatosis; SEM, standard error of the mean.

Patients underwent up to 39 apheresis procedures using either the Optia or Alyx system. Inclusion criteria of the patients were as follows: weight > 50 kg, Hb > 12.5 g/dL, hematocrit (Hct) > 37%, age 18–80, and hyperferritinemia. Exclusion criteria were active malignancy, severe heart failure, serious cardiac arrhythmias, and uncontrolled epilepsy.

### 2.2 Study Design

The study is a retrospective observational study. Laboratory values were measured both before and after RBC depletion (Hb, Hct, platelets) by using a Sysmex XP‐300 hematology analyzer (Sysmex Corporation, Kobe, Japan) or only after RBC depletion (serum ferritin SF, transferrin saturation TS). In addition, blood pressure and pulse of the patients were determined before and after each procedure.

Data from a total of 186 procedures, including the duration of procedures, the volume of processed blood, the volume of anticoagulant and saline infused to the patients, were obtained for analysis. All apheresis procedures were performed in our institution.

### 2.3 Therapeutic RBC Apheresis

For RBC apheresis, we either used the automated RBC depletion program of the Optia or the Alyx device. For Optia procedures, the automated depletion program was selected with the following settings: fluid balance 100% and target Hct between 5% and 6% (mean 5.6%) below the initial value. The Optia requires two venous accesses; meanwhile, there is a single needle access available. The removed RBC volume was replaced with saline, and the extracorporeal volume is about 175 mL. The Optia is a therapeutic device designed with patient safety and flexibility in mind. Here, the RBC layer is separated in a concentrated manner, but not always to maximum density as the machine regulates in real time according to blood flow, patient Hct, and exchange volume. Optia uses the rotating centrifuge chamber with continuous control (optical sensor and software—Automated Interface Management AIM) [7].

The Alyx apheresis machine was originally developed to collect 2 units of RBC within one single procedure. It works with a fixed separation that is calibrated for double RBCs and is optimized to produce standardized packed RBCs in the collection bag that can be easily processed further. Due to this reason, the Alyx collects very dense packed RBCs with a constant Hct. We used it as a method of phlebotomy for patients with elevated ferritin. The Alyx was set for a collection of 360 mL RBC. The removed RBC volume was determined from the absolute RBC volume counter displayed on the Alyx component collection system. It is a single needle procedure with multiple collection and return cycles. The patient extracorporeal volume is higher (about 300–400 mL) compared to the Optia (175 mL). There are short collection cycles with a certain volume of blood collected per cycle and saline injection at each return cycle, all based on patient height, weight and Hb status. It is designed for healthy donors, and therefore a standardized volume, in our case 360 mL of RBC, is drawn away in each procedure.

In both proceedings, no plasma or platelet components were collected additionally. Oral calcium supplementation was not routinely given, but was provided on request to treat symptoms of citrate toxicity. The anticoagulant used was Anticoagulant Citrate Dextrose Solution (ACD‐A); volume loss was automatically replaced with saline by the system.

### 2.4 Statistical Analysis

Data are presented as arithmetic mean ± standard error of the mean (SEM). To analyze variables of interest the Mann–Whitney U‐Test was applied. Bonferroni Holm was used to account for multiplicity. A *p* value < 0.05 was assumed to be significant. Analyses were performed using the statistic software R (Version 4.1.3) and Excel [8].

To investigate the SF reduction, it was measured at the next consultation after RBC depletion. The intervals between measurements had to be between one and 7 weeks. The runs that met these criteria were included in the subgroups. Longer intervals between RBC depletions were excluded.

## 3 Results

### 3.1 Patient Characteristics

**FIGURE 1:**
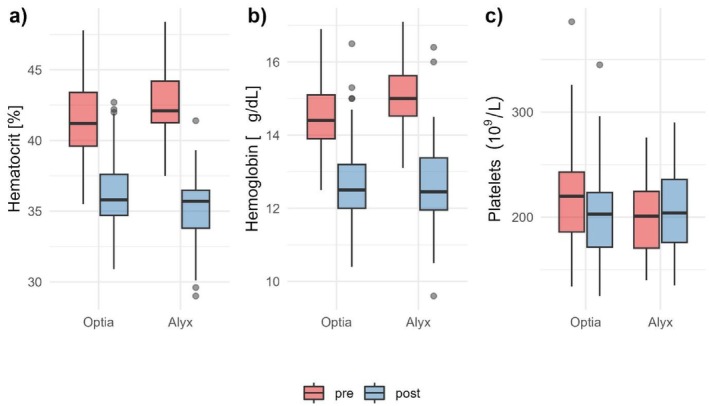
Pre‐ und postapheresis characteristics. Boxplots illustrating (a) Hct [%], (b) Hb [g/dL], and (c) Platelet count [10^9^/L] measured before (pre, red) and after (post, blue) RBC depletion performed with the Optia and Alyx systems.

There were 36 patients with a total of 143 runs with the Optia and 20 patients with a total of 43 runs with the Alyx. Patient characteristics as mean age (50.6 ± 2.18 years in the Optia and 54.8 ± 2.7 in the Alyx), weight (91.9 ± 2.4 kg and 93.8 ± 3.7 kg), height (180.6 cm and 181.3 cm) and total blood volume (5730 ± 102.9 mL and 5839 ± 155.9 mL) hardly differed between Optia and Alyx (*p* > 0.05). The majority of the patients were male (35 vs. 20 males), with only one female treated with the Optia. The main diagnosis in both groups was HH (17 on Optia, 13 on Alyx), followed by DIOS (13 vs. 6) and hyperferritinemia (4 vs. 1). One patient was depleted with the Optia due to secondary polyglobulia and one due to iatrogenic iron overload. Pre‐ and post‐apheresis data are shown in Figure 1.

### 3.2 Parameters

**FIGURE 2:**
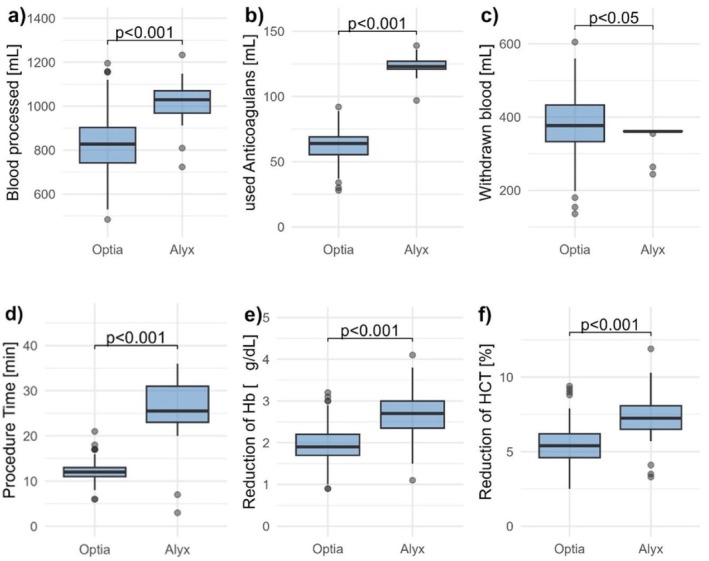
Parameters and *p* values of the two apheresis systems. Boxplots comparing (a) processed blood volume [mL], (b) anticoagulant volume used [mL], (c) withdrawn blood volume [mL], (d) procedure time [min], (e) reduction of Hb [g/dL], and (f) reduction of Hct [%] between procedures performed with the Optia and Alyx systems.

In a subgroup (124 procedures in Optia, 39 procedures in Alyx) we examined SF reduction by defining the SF level on the day of depletion as SF pre and the level at the next consultation as SF post. In most cases depletions were performed at two to 4 weeks intervals depending on SF and Hb levels. A higher SF reduction was observed in patients treated with Optia (117.8 ± 11.8 μg/L after 20 ± 0.8 days vs. 82.6 ± 15.8 μg/L with Alyx after 22 ± 1.2 days), but this reduction was not statistically significant (*p* = 0.3). We observed a greater reduction in Hct values after depletion in the Alyx, by 7.5% ± 0.3%, compared to 5.0% ± 0.2% in the Optia. Similarly, the Hb levels after depletion were 2.7 ± 0.1 g/dL lower in the Alyx and only 1.8 ± 0.1 g/dL lower in the Optia, as shown in Figure 2. Platelet levels were actually slightly higher in the Alyx than at baseline, while they were reduced by 13 800 ± 2370 in the Optia, as shown in Figure 1.

On average, 1021 ± 12.8 mL of blood were processed with the Alyx, and only 817 ± 12.1 mL with the Optia. The significantly shorter time for the Optia, 12 ± 0.2 min for a run compared to 26 ± 1 min with the Alyx, also means that almost half as much anticoagulant was used (63 ± 0.9 mL compared to 124 ± 1.1 mL). It should be noted that the Alyx is a single‐arm procedure.

With the Optia, slightly more blood is taken from the patient than with the Alyx (376 ± 6.8 vs. 356 ± 3.6 mL). All parameters and *p* values are shown in Table 2 and Figure 2.

**TABLE 2:** Variables of 186 apheresis procedures^**a**^.

Parameter	Instruments		*p*
	Optia	Alyx	
Number of procedures	143	43	
Procedure	2‐arm procedure^c^	1‐arm procedure	
**Procedure time in min**	**12 ± 0.2**	**26 ± 1**	**< 0.001**
**Blood processed in mL**	**817 ± 12.1**	**1021 ± 12.8**	**< 0.001**
Withdrawn blood in mL	376.2 ± 6.8	356.3 ± 3.6	< 0.05
**Saline replacement in mL**	**345 ± 6.1**	**395.3 ± 7.5**	**< 0.001**
**ACD‐A in mL**	**62.8 ± 0.9**	**123.5 ± 1.1**	**< 0.001**
Hct pre in %	41.6 ± 0.2	42.5 ± 0.4	> 0.05
Hct post in %	36.3 ± 0.2	35.9 ± 0.6	> 0.05
**Reduction of Hct in %**	**4.96 ± 0.22**	**7.53 ± 0.28**	**< 0.001**
Hb pre in g/dL	14.5 ± 0.1	15.1 ± 0.2	> 0.05
Hb post in g/dL	12.7 ± 0.1	12.6 ± 0.2	> 0.05
**Reduction of Hb in g/dL**	**1.78 ± 0.07**	**2.68 ± 0.11**	**< 0.001**
PLT pre/μL	219 000 ± 3400	199 500 ± 3430	> 0.05
PLT post/μL	199 000 ± 3100	201 000 ± 5600	> 0.05
**Reduction of PLT**	**13 780 ± 2370**	**‐2520 ± 1820**	**< 0.001**
**SF pre in μg/L** ^b^	**1038.9 ± 92.0**	**473.3 ± 84.2**	**< 0.001**
**SF post in μg/L** ^b^	**921.0 ± 89.4**	**390.7 ± 90.3**	**< 0.001**
Reduction of SF in μg/L^b^	117.8 **± 11.8**	82.59 **± 15.8**	> 0.05
**Discard bag in mL**	**405.1 ± 7**	**441.9 ± 12.8**	**< 0.001**
Initial Hct in %	41.6 ± 0.2		
Target Hct in %	36.0 ± 0.2		
Extracorporeal volume in mL	⁓175	⁓300–400	

*Note:* Bold values are statistically significant (*p* < 0.001).

Abbreviations: ACD‐A, anticoagulant citrate dextrose solution, formula A; Hb, hemoglobin; Hct, hematocrit; PLT, platelets; SF, serum ferritin.

^a^ Data are shown as mean ± standard error of the mean (SEM).

^b^ Subgroup of 124 (Optia) and 39 (Alyx) procedures.

^c^ 1‐arm procedure not available in Europe before 2024.

### 3.3 Side Effects and Technical Problems

Furthermore, we observed more side effects or technical problems with the Alyx: one patient reported two times mild dizziness, two procedures in two different patients had to be aborted due to technical problems with the optical sensor of the device and due to kinking alarms, respectively.

One procedure had to be stopped after 23 min because the blood flow could not be maintained due to the patient's poor vein status. During the Optia procedures, only 1 patient reported vasovagal reactions shortly after initiating intravenous access.

Patients were only asked about side effects during the procedure and after 30 min observation time. Delayed side effects after leaving the hospital were not recorded.

## 4 Discussion

To our knowledge, our study is the first to compare the performance of RBC depletion between Optia and Alyx.

The main characteristics of the Alyx are its portability, thanks to its small size, and its short collection cycles, with an extracorporeal volume of around 300–400 mL [9, 10, 11]. Advantage of the Alyx includes the single needle procedure, especially for patients with poor veins, and the favorable cost.

Since 2024 a single needle option for the Spectra Optia is also available in Europe with software version 12.0. At the time of our study, this option was not yet available.

Another difference between the two systems relates to the instrument settings, which are different in each case. With the Optia, the target Hct is entered (approx. 6% below the patient's initial Hct) and the apheresis device uses this data to calculate the amount of red cells removed and the replacement depletion.

The Alyx, on the other hand, removes a fixed volume of RBCs depending on the device settings, in our case 360 mL—regardless of the patient's Hct. This also explains the higher Hct reduction with the Alyx.

In general, RBC depletion offers a good alternative to conventional phlebotomy to achieve an effective RBC reduction and in consequence an effective iron and ferritin reduction. Other studies showed that RBC apheresis removed twice as much iron and RBCs when compared to phlebotomy [12, 13].

We had an average RBC depletion interval of 21 days with both devices. Högler et al. showed that after the production of double RBC concentrates, SF decreases after 7–30 days, with a nadir after 1 month of donation and then rises again [14]. Despite the higher Hct reduction with Alyx, there was no statistically significant difference in the SF reduction. The reduction in SF is not linear with the reduction in Hct and serum iron levels. Eisfeld et al. also could observe a plateau after an initial steady decline in SF followed by a second decline under further phlebotomy [15]. One reason for the higher SF decrease in the Optia could be the higher initial SF levels in patients treated with Optia (1038.9 ± 92.0 μg/L vs. 473.3 ± 84.2 μg/L). This correlation was also observed from Eisfeld et al. [15].

Therefore, factors other than the reduction in Hct and the associated reduction in iron can influence SF levels. It is worth mentioning that SF is an acute‐phase protein [16] and obesity, higher alcohol and heme iron consumption in males, and age in females were associated with iron overload [17, 18]. Overall, it can be said that an efficient reduction in SF was achieved with both devices.

A further advantage of RBC depletion is the preservation of plasma proteins, platelets, WBCs, and coagulation factors by returning blood to the patient. Therefore, both procedures could be used in patients with low platelet levels or hypoproteinemia [6].

The platelet count in the Alyx even increased slightly (Figure 1). These observations do not go along with Schooneman's publication in 2005, which found a decrease in platelets after the procedure [7]. The higher platelet count shortly after the RBC depletion may be due to changes in rheology. The Optia also showed only a slight loss of platelets and WBCs after RBC depletion.

In general, RBC depletion was well tolerated with both procedures. We observed more side effects with the Alyx than with the Optia, although the side effects can be classified as very mild. In 2016, Keshelashvili et al. described hematomas and mild vasovagal reactions as the most common side effects with the Alyx [11], while we rather observed technical problems and mild dizziness in our patients.

A comparison of the costs shows that the purchase price of Optia was twice that of Alyx in our case. A disposable set of the Optia was three times more expensive than an Alyx set. Depletion procedures with Optia will primarily take place in a hospital‐based apheresis service, so if an Optia apheresis device is already available at a centre, RBC depletion with this system will be an option. The low side effect rate of the Optia and the individual HCT adjustment for certain patients are advantageous. With Optia, no RBC unit can be produced; the collected RBCs must be discarded.

The Alyx is no longer used in Europe due to a sharp decline in autologous blood donation and a small discontinuation market. In the USA, the Alyx is regularly used for the donation of double RBC units, mainly in a donor center or mobile blood drives. But Choe et al. also used the Alyx for RBC depletion for the treatment of erythrocytosis [19].

One way to generate more RBC units is to produce double RBC units from donors with HH. According to Leitman et al.a large number of additional RBC units can be obtained from donors with HH [20]. In many countries, HH patients are approved to donate blood, however, in others, they are deferred due to concerns about safety, compliance and cost [21]. In Austria, donors with HH have been able to donate under certain conditions since 2008. The production of a double RBC unit as a “side effect” of this therapy is a major advantage of the Alyx.

We have shown that RBC depletion is a suitable and tolerable method for treating patients with HH. Furthermore, the costs of RBC depletion compared to conventional phlebotomy are relativized by the shorter treatment time shown by Rombrout‐Setrienkova [22].

In addition, the economic impact should also be taken into account. The fact that the patient visits the clinic less often means that he or she loses fewer days at work. From an economic point of view, the higher cost of RBC depletion can be offset [22].

The limitations to our work are the small size of our patient cohort and the retrospective study design.

## 5 Conclusion

We recommend the Alyx as well as the Optia for RBC depletion in patients with hyperferritinemia. The advantages of the Alyx are the lower costs and the easy portability. In certain cases, the Alyx can also be used to obtain double RBC units from HH patients, providing an additional benefit to the therapy. The Optia, on the other hand, has a shorter procedure time, a smaller extracorporeal volume, lower anticoagulant consumption, and a lower complication rate. The Optia has the disadvantage of being more expensive in terms of both the initial cost and the set price. However, if an Optia apheresis device is already available and used for other therapies, the Optia is certainly a good option for performing RBC depletions and enables a precise Hct adjustment.

## Funding

The authors have nothing to report.

## Disclosure

Permission to reproduce material from other sources: This manuscript does not include any third‐party material requiring permission for reproduction.

## Ethics Statement

The study was conducted in accordance with the declaration of Helsinki and its later amendments. Anonymous data analyses were performed with permission of the local ethics committee (approval number 1022/2022).

## Consent

Patient consent was waived by the local ethics committee due to the retrospective nature of the study and the use of anonymized data.

## Conflicts of Interest

The authors declare no conflicts of interest.

## Data Availability

The data that support the findings of this study are available from the corresponding author upon reasonable request.
